# Standards, Options and Recommendations: list of publications

**DOI:** 10.1054/bjoc.2001.1772

**Published:** 2001-05


British Journal of Cancer (2001) **84**(Supplement 2), 86–91
© 2001 FNCLCC
doi: 10.1054/ bjoc.2001.1772, available online at http://www.idealibrary.com on IDEAL®

Appendix

### Standards, Options and Recommendations: list of publications

### 1. METHODOLOGY

### 1.1 Methodology in the development of SOR

Fervers B, Blanc-Vincent MP, Bonichon F, Farsi F, Labrèze L, Lehmann M, Bey P, Philip T, Renaud-Salis JL, Sarradet A and Theobald S (1998) Méthodologie d'élaboration des standards, options et recommandations diagnostiques et thérapeutiques en cancérologie de la Fédération Nationale des Centres de Lutte Contre le Cancer. In: Fédération Nationale des Centres de Lutte Contre le Cancer, ed. *Recommandations pour la pratique clinique en cancérologie* [CD-Rom]. FNCLCC, John Libbey EUROTEXT: Paris

Fervers B, Bonichon F, Demand F, Heron JF, Mathoulin S, Philip T, Renaud-Salis JL, Toma C and Bey P (1995) Méthodologie de développement des standards, options et recommandations diagnostiques et thérapeutiques en cancérologie. *Bull Cancer* **82**: 761–767

### 1.2 Review and critical analysis of the literature

Bonichon F, Courtial F and Blanc-Vincent MP (1998) Revue de la littérature, recherche d'informations et lecture critique d'articles en cancérologie. *Bull Cancer* **85**: 867–885

Bonichon F and Courtial F (1995) Revue de la littérature, recherche d'informations et lecture critique d'articles en cancérologie. *Bull Cancer* **82**(10): 768–779

### 2. CANCER IN THE ADULT

### 2.1 Gynaecological malignancies

#### 2.1.1 Non-metastatic breast cancer

Mauriac L, Blanc-Vincent MP, Luporsi E, Cutuli B, Fourquet A, Garbay JR, Giard S, Spyratos F, Zafrani B and Dilhuydy JM (2000) Standards, options et recommandations: hormonothérapie dans les cancers du sein non métastatiques. *Bull Cancer* **87**: 469–490

Mauriac L, Asselain B, Blanc-Vincent MP, Cutuli B, Dilhuydy JM, Fourquet A, Garbay JR, Luporsi E, Spyratos F and Zafrani B (1996) Fédération Nationale des Centres de Lutte contre le Cancer, Standards, options et recommandations. *Cancers du sein non métastatiques, Vol. 3*. Amette Blackwell: Paris

Mauriac L, Asselain B, Blanc-Vincent MP, Cutuli B, Dilhuydy JM, Fourquet A, Garbay JR, Luporsi E, Spyratos F and Zafrani B (1998) Standards, options et recommandations: Cancers du sein non métastatiques. In: Fédération Nationale des Centres de Lutte Contre le Cancer, ed. *Recommandations pour la pratique clinique en cancérologie* [CD-Rom]. FNCLCC, John Libbey EUROTEXT: Paris

#### 2.1.2 Cancer of the cervix

Haie-Meder C, Fervers B, Chauvergne J, Fondrinier E, Lhommé C, Guastalla JP and Resbeut M (1999) Radiochimiothérapie concomitante dans les cancers du col de l'utérus: analyse critique des données et mise à jour des standards, options et recommandations. *Bull Cancer* **86**: 829–841

Resbeut M, Fondrinier E, Fervers B, Asselain B, Basuyau JP, Brémond A, Castaigne D, Chauvergne J, Dubois JB, Haie-Meder C, Houvenaeghel G, Lartigau E, Leblanc E, Sarradet A, Sastre-Garau X and Ternier F (1999) Fédération Nationale des Centres de Lutte Contre le Cancer, Société Française d'Oncologie Gynécologique. Standards, options et recommandations. *Cancers invasifs du col utérin (stades non métastatiques)*, vol.9.John Libbey EUROTEXT: Paris

Resbeut M, Fondrinier E, Fervers B, Asselain B, Basuyau JP, Brémond A, Castaigne D, Chauvergne J, Dubois JB, Haie-Meder C, Houvenaeghel G, Lartigau E, Leblanc E, Sarradet A, Sastre-Garau X and Ternier F (1998) Standards, options et recommandations pour la prise en charge des patientes atteintes de cancers invasifs du col utérin (stades non métastatiques). In: Fédération Nationale des Centres de Lutte Contre le Cancer, ed. *Recommandations pour la pratique clinique en cancérologie* [CD-Rom]. Paris: FNCLCC, John Libbey EUROTEXT.

#### 2.1.3 Ovarian cancer

Kerbrat P, Fervers B, Basuyau JP, Cohen-Solal C, Dauplat J, Duvillard P, Guastalla JP, Lhommé C, Thomas L and Tournemaine N, Fédération Nationale des Centres de Lutte Contre le Cancer, Société Française d'Oncologie Gynécologique. *Standards, options et recommandations. Tumeurs épithéliales malignes de l'ovaire*. Paris: John Libbey EUROTEXT, 1998; vol.4

Kerbrat P, Fervers B, Basuyau JP, Cohen-Solal C, Dauplat J, Duvillard P, Guastalla JP, Lhommé C, Thomas L and Tournemaine N (1998) Standards, options et recommandations pour la prise en charge initiale des patientes atteintes de tumeurs épithéliales malignes de l'ovaire. In: Fédération Nationale des Centres de Lutte Contre le Cancer, ed. *Recommandations pour la pratique clinique en cancérologie* [CD-Rom]. Paris: FNCLCC, John Libbey EUROTEXT

### 2.2 Gastrointestinal malignancies

#### 2.2.1 Hepatocellular carcinoma

Giovannini M, Elias D, Monges G, Raoul JL, Rougier P, Sarradet A and Toma C (1998) Standards, options et recommandations pour la prise en charge des patients atteints de carcinomes hépatocellulaires. In: Fédération Nationale des Centres de Lutte Contre le Cancer, ed. *Recommandations pour la pratique clinique en cancérologie* [CD-Rom]. Paris: FNCLCC, John Libbey EUROTEXT

Giovannini M, Elias D, Monges G, Raoul JL, Rougier P and Toma C (1995) Standards, options et recommandations pour la prise en charge des patients atteints de carcinomes hépato-cellulaires. In: Fédération Nationale des Centres de Lutte Contre le Cancer, ed. *Cancers digestifs: Cancers de l'oesophage, carcinome hépato-cellulaire, cancer du côlon*. Paris: Arnette Blackwell, vol.2:39–83

#### 2.2.2 Colon cancer

Conroy T and Adenis A (1998) pour le groupe de travail SOR «cancer du côlon» Standards, options et recommandations pour la surveillance après traitement d'un cancer du côlon. *Bull Cancer* **85**: 152–159

Adenis A, Conroy T, Lasser P, Merrouche Y, Monges G, Rivoire M, Rougier P, Ruggieri-Pignon S, Sarradet A and Toma C (1998) Standards, options et recommandations pour la prise en charge des patients atteints de cancer du côlon. In: Fédération Nationale des Centres de Lutte Contre le Cancer, ed. *Recommandations pour la pratique clinique en cancérologie* [CD-Rom]. Paris: FNCLCC, John Libbey EUROTEXT

Adenis A, Conroy T, Lasser P, Merrouche Y, Monges G, Rivoire M, Rougier P, Ruggieri-Pignon S and Toma C (1995) Standards, options et recommandations pour la prise en charge des patients atteints de cancer du côlon. In: Fédération Nationale des Centres de Lutte Contre le Cancer, ed. *Cancers digestifs: cancer de l'oesophage, carcinome hépato-cellulaire, cancer du côlon*. Paris: Amette Blackwell, vol.2:85–160

#### 2.2.3 Oesophageal cancer

Seitz JF, François E, Jacob JH, Ollivier JM, Rougier P, Roussel A, Sarradet A and Toma C (1998) Standards, options et recommandations pour la prise en charge des patients atteints de cancer de l'oesophage. In: Fédération Nationale des

Centres de Lutte Contre le Cancer, ed. *Recommandations pour la pratique clinique en cancérologie* [CD-Rom]. Paris: FNCLCC, John Libbey EUROTEXT.

Seitz JF, François E, Ollivier JM, Rougier P, Roussel A and Toma C (1995) Standards, options et recommandations pour la prise en charge des patients atteints de cancer de l'oesophage. In: Fédération Nationale des Centres de Lutte Contre le Cancer, ed. *Cancers digestifs: cancer de l'oesophage, carcinome hépato-cellulaire, cancer du côlon*. Paris: Arnette Blackwell, vol. 2: 1–37

### 2.2.4 Cancer of the rectum

Bécouarn Y, Blanc-Vincent MP, Lasser P, Dubois JB, Ducreux M, Giovannini M, Rougier P and et entretien avec Y. Bécouam (1999) Standards, Options et Recommandations pour la prise en charge des patients atteints d'adénocarcinome primitif du rectum. *Presse Med* **28**: 1367–1377

Bécouam Y, Blanc-Vincent MP, Lasser P, Dubois JB, Ducreux M, Giovannini M and Rougier P (1998) Standards, options et recommandations pour la prise en charge des patients atteints d'adénocarcinome primitif du rectum. In: Fédération Nationale des Centres de Lutte Contre le Cancer, ed. *Cancers digestifs II: pancréas et rectum*. Paris: John Libbey EUROTEXT, vol.5: 79–229.

Bécouam Y, Blanc-Vincent MP, Lasser P, Dubois JB, Ducreux M, Giovannini M and Rougier P (1998) Standards, options et recommandations pour la prise en charge des patients atteints d'adénocarcinome primitif du rectum. In: Fédération Nationale des Centres de Lutte Contre le Cancer, ed. *Recommandations pour la pratique clinique en cancérologie* [CD-Rom]. Paris: FNCLCC, John Libbey EUROTEXT

### 2.2.5 Cancer of the pancreas

Ducreux M, François E, Saint-Aubert B, NGuyen TD, Sarradet A and Rougier P (1998) Standards, options et recommandations pour la prise en charge des patients atteints de cancer du pancréas. In: Fédération Nationale des Centres de Lutte Contre le Cancer, ed. *Cancers digestifs II: pancréas et rectum*. Paris: John Libbey EUROTEXT vol.5: 3–77

Ducreux M, François E, Saint-Aubert B, NGuyen TD, Sarradet A and Rougier P (1998) Standards, options et recommandations pour la prise en charge des patients atteints de cancer du pancréas. In: Fédération Nationale des Centres de Lutte Contre le Cancer, ed. *Recommandations pour la pratique clinique en cancérologie* [CD-Rom]. Paris: FNCLCC, John Libbey EUROTEXT

### 2.3 Haematological malignancies

### 2.3.1 Hodgkin's disease

Fermé C, Cosset JM, Fervers B, Sebban C, Cutuli B, Henry-Amar M, Stines J, Giammarile F, Bey P, Carella AM and Philip T Groupe d'Etude des Lymphomes de l'Adulte, et entretien avec le Dr C. Fermé (1999) Standards, options et recommandations pour la prise en charge des patients adultes atteints de la maladie de Hodgkin. *Presse Med* **28**(40): 2233–2245

Fermé C, Cosset JM, Sebban C, Fervers B, Cutuli B, Henry-Amar M, Stines J, Giammarile F, Bey P, Carella AM and Philip T, Fédération Nationale des Centres de Lutte Contre le Cancer, Groupe d'Etude des Lymphomes de l'Adulte (1999) Standards, options et recommandations. Maladie de Hodgkin chez l'adulte. In: Fédération Nationale des Centres de Lutte Contre le Cancer, ed. *Maladie de Hodgkin chez l'adulte. Lymphomes malins non hodgkiniens cérébraux primitifs en dehors de l'infection par VIH*. Paris: John Libbey EUROTEXT, vol.7: 1–166

Fermé C, Cosset JM, Sebban C, Fervers B, Cutuli B, Henry-Amar M, Stines J, Giammarile F, Bey P, Carella AM and Philip T, Groupe d'Etude des Lymphomes de l'Adulte (1998) Standards, options et recommandations pour la prise en charge des patients adultes atteints de la maladie de Hodgkin. In: Fédération Nationale des Centres de Lutte Contre le Cancer, ed. *Recommandations pour la pratique clinique en cancérologie* [CD-Rom]. Paris: FNCLCC, John Libbey EUROTEXT

### 2.3.2 Cerebral lymphoma

Blay JY, Farsi F, Carrie C, Biron P, Fervers B and Philip T (1999) Standards, options et recommandations. Lymphomes malins non hodgkiniens cérébraux primitifs en dehors de l'infection par VIH. In: Fédération Nationale des Centres de Lutte Contre le Cancer, ed. *Maladie de Hodgkin chez l'adulte. Lymphomes malins non hodgkiniens cérébraux primitifs en dehors de l'infection par VIH*. Paris: John Libbey EUROTEXT, vol.7: 169–217

Blay JY, Farsi F, Carrie C, Biron P, Fervers B and Philip T (1998) Standards, options et recommandations pour la prise en charge des patients atteints de lymphomes malins non hodgkiniens cérébraux primitifs en dehors de l'infection par VIH. In: Fédération Nationale des Centres de Lutte Contre le Cancer, ed. *Recommandations pour la pratique clinique en cancérologie* [CD-Rom]. Paris: FNCLCC, John Libbey EUROTEXT

### 2.4 Tumours of the skin and soft tissue

### 2.4.1 Soft-tissue sarcoma

Bui BN, Blay JY, Bonichon F, Busson A, Carrie C, Chauvel P, Coindre JM, Cuisenier J, Delannes M, Kantor G, Kind M, Lagarde P, Le Cesne A, Ollivier L, Peiffert D, Rivoire M, Stöckle E and Terrier P (1995) Standards, options et recommandations pour la prise en charge des patients adultes atteints de sarcomes des tissus mous. In: Fédération Nationale des Centres de Lutte Contre le Cancer, ed. *Sarcomes des tissus mous et ostéosarcomes*. Paris: Arnette Blackwell, vol.1: 1–113

Bui BN, Blay JY, Bonichon F, Busson A, Carrie C, Chauvel P, Coindre JM, Cuisenier J, Delannes M, Kantor G, Kind M, Lagarde P, Le Cesne A, Ollivier L, Peiffert D, Rivoire M, * E and Terrier P (1998) Standards, options et recommandations pour la prise en charge des patients adultes atteints de sarcomes des tissus mous. In: Fédération Nationale des Centres de Lutte Contre le Cancer, ed. *Recommandations pour la pratique clinique en cancérologie* [CD-Rom]. Paris: FNCLCC, John Libbey EUROTEXT

### 2.4.2 Malignant melanoma

Négrier S, Fervers B, Bailly C, Beckendorf V, Cupissol D, Doré JF, Dorval T, Garbay JR, Vilmer C and et entretien avec le Docteur S. Négrier (2000) Standards, Options et Recommandations pour la prise en charge des patients atteints de mélanome cutané. *Presse Med* **29**: 1317–1326

Négrier S, Fervers B, Bailly C, Beckendorf V, Cupissol D, Doré JF, Dorval T, Garbay JR and Vilmer C (2000) Standards, options et recommandations pour la prise en charge des patients atteints de mélanome cutané. *Bull Cancer* **87**(2): 173–182

Négrier S, Fervers B, Bailly C, Beckendorf V, Cupissol D, Doré JF, Dorval T, Garbay JR and Vilmer C, (1998) Fédération Nationale des Centres de Centres de Lutte Contre le Cancer *Standards, Options et Recommandations: Mélanome cutané*. Paris: John Libbey EUROTEXT, vol.6

Négrier S, Fervers B, Bailly C, Beckendorf V, Cupissol D, Doré JF, Dorval T, Garbay JR and Vilmer C (1998) Standards, options et recommandations pour la prise en charge des patients atteints de mélanome cutané. In: Fédération Nationale des Centres de Lutte Contre le Cancer, ed. *Recommandations pour la pratique clinique en cancérologie* [CD-Rom]. Paris: FNCLCC, john Libbey EUROTEXT

### 2.5 Cancers of the head and neck

### 2.5.1 Cancer of the salivary glands

Bensadoun RJ, Blanc-Vincent MP, Chauvel P, Dassonville O and Demard F (1998) Standards, options et recommandations pour les patients atteints de tumeurs malignes des glandes salivaires (lymphomes inclus). In: Fédération Nationale des Centres de Lutte Contre le Cancer, ed. *Recommandations pour la pratique clinique en cancérologie* [CD-Rom]. Paris: FNCLCC, John Libbey EUROTEXT

### 2.5.2 Cancers of the oral cavity

Brugère J, Brunin F, Farsi F, Gourmet R, Jaulerry C, Peiffert D and Rodriguez J (1998) Standards, options et recommandations pour la prise en charge des patients atteints de carcinomes épidermoïdes de la cavité buccale. In: Fédération Nationale des Centres de Lutte Contre le Cancer, ed. *Recommandations pour la pratique clinique en cancérologie* [CD-Rom]. Paris: FNCLCC, John Libbey EUROTEXT

### 2.5.3 Cancers of the oropharynx

Renaud-Salis JL, Blanc-Vincent MP, Brugère J, Demard F, Faucher A, Gory-Delabaere G and Pinsolle J (1999) Standards, options et recommandations pour le diagnostic, le traitement et la surveillance des cancers épidermoïdes de l'oropharynx. *Bull Cancer* **86**(6): 550–572.

Renaud-Salis JL, Blanc-Vincent MP, Brugère J, Demard F and Faucher A (1998) Standards, options et recommandations pour le diagnostic, le traitement et la surveillance des cancers épidermoïdes de l'oropharynx. In: Fédération Nationale des Centres de Lutte Contre le Cancer, ed. *Recommandations pour la pratique clinique en cancérologie* [CD-Rom]. Paris: FNCLCC, John Libbey EUROTEXT.

© 2001 FNCLCC

### 2.6 Others

#### 2.6.1 Renal-cell carcinoma

Beckendorf V, Bladou F, Farsi F, Kaemmerien P, Négrier S, Phillip T and Terrier-Lacombe MJ (2000) Standards, options et recommandations pour la radiothérapie du cancer du rein. *Cancer Radiother* **4**: 60–75.

Beckendorf V, Bladou F, Farsi F, Kaemmerien P, Négrier S, Philip T, Terrier-Lacombe MJ (1998) Standards, options et recommandations pour la prise en charge des patients atteints de cancers du rein. In: Fédération Nationale des Centres de Lutte Contre le Cancer, ed. *Recommandations pour la pratique clinique en cancérologie* [CD-Rom]. Paris: FNCLCC, John Libbey EUROTEXT

#### 2.6.2 Thymic tumours

Ruffié P, Gory-Delabaere G, Fervers B, Lehmann M, Regnard JF and Resbeut M (1999) Standards, options et recommandations pour la prise en charge des patients atteints de tumeurs épithéliales du thymus. *Bull Cancer* **86**: 365–384.

Ruffié P, Bretel JJ, Lagrange JL and Lehmann M (1998) Standards, options et recommandations pour la prise en charge des patients atteints de tumeurs épithéliales du thymus. In: Fédération Nationale des Centres de Lutte Contre le Cancer, ed. *Recommandations pour la pratique clinique en cancérologie* [CD-Rom]. Paris: FNCLCC, John Libbey EUROTEXT

#### 2.6.2 Malignant mesothelioma

Ruffié P, Lehmann M, Galateau-Sallé F, Lagrange JL and Pairon JC (2000) Standards, options et recommandations pour la prise en charge des patients atteints de mésothéliome malin de la plèvre. *Presse Med* A paraître.

Ruffié P, Lehmann M, Galateau-Sallé F, Lagrange JL and Pairon JC (1998) Standards, options et recommandations pour la prise en charge des patients atteints de mésothéliome malin de la plèvre. *Bull Cancer* **85**: 545–561

Ruffié P, Lehmann M, Galateau-Sallé F, Lagrange JL and Pairon JC (1998) Standards, options et recommandations pour la prise en charge des patients atteints de mésothéliome malin de la plèvre. In: Fédération Nationale des Centres de Lutte Contre le Cancer, ed. *Recommandations pour la pratique clinique en cancérologie* [CD-Rom]. Paris: FNCLCC, John Libbey EUROTEXT

### 3. PAEDIATRIC MALIGNANCIES

### 3.1 Cancer of bone and soft tissue

#### 3.1.1 Osteosarcoma

Philip T, Blay JY, Brunat-Mentigny M, Carrie C, Chauvot P, Farsi F, Fervers B, Gentet JC, Giammarile F, Kohler R, Mathoulin S, Patricot LM and Thiesse P (1999) Standards, options et recommandations pour le diagnostic, le traitement, et la surveillance de l'ostéosarcome. *Bull Cancer* **86**: 159–176

Brunat-Mentigny M, Blay JY, Carrie C, Chauvot P, Fervers B, Gentet JC, Kohler R, Mathoulin S, Patricot L, Philip T and Thiesse P (1995) Standards, options et recommandations pour le diagnostic, le traitement et la surveillance de l'ostéosarcome. In: Fédération Nationale des Centres de Lutte Contre le Cancer, ed. *Sarcomes des tissus mous et ostéosarcomes* Paris: Arnette Blackwell, vol.1: 115–168

Philip T, Blay JY, Brunat-Mentigny M, Carrie C, Chauvot P, Farsi F, Fervers B, Gentet JC, Giammarile F, Kohler R, Mathoulin S, Patricot L, Thiesse P, Fédération Nationale des Centres de Lutte Contre le Cancer, Société Française d'Oncologie Pédiatrique (1998) Standards, options et recommandations pour le diagnostic, le traitement et la surveillance de l'ostéosarcome. In: Fédération Nationale des Centres de Lutte Contre le Cancer, ed. *Recommandations pour la pratique clinique en cancérologie* [CD-Rom]. Paris: FNCLCC, John Libbey EUROTEXT.

#### 3.1.2 Rhabdomyosarcoma

Pinkerton R, Sommelet D, Brunat-Mentigny M, Farsi F, Martel I, Philip T, Ranchère-Vince D, Thiesse P, Fédération Nationale des Centres de Lutte Contre le Cancer, Société Française d'Oncologie Pédiatrique (1998) Standards, options et recommandations pour la prise en charge des patients atteints de rhabdomyosarcomes et autre tumeurs mesenchymateuses malignes non-rhabdomyosarcomes de l'enfant. *Bull Cancer* **85**: 1015–1042.

Pinkerton R, Sommelet D, Brunat-Mentigny M, Farsi F, Martel I, Phillip T, Ranchère-Vince D, Thiesse P, Fédération Nationale des Centres de Lutte Contre le Cancer, Société Française d'Oncologie Pédiatrique (1998) Standards, options et recommandations pour la prise en charge des patients atteints de rhabdomyosarcomes et autre tumeurs mesenchymateuses malignes non-rhabdomyosarcomes de l'enfant. In: Fédération Nationale des Centres de Lutte Contre le Cancer, ed. *Recommandations pour la pratique clinique en cancérologie* [CD-Rom]. Paris: FNCLCC, John Libbey EUROTEXT.

### 3.2 Tumours of the central nervous system

#### 3.2.1 Medulloblastoma

Doz F, Alapetite C, Chastagner P, Choux M, Gaboriaud G, Pontvert D, Sarradet A, Fédération Nationale des Centres de Lutte Contre le Cancer, Société Française d'Oncologie Pédiatrique (1999) Standards, options et recommandations. Médulloblastome de l'enfant. In: Fédération Nationale des Centres de Lutte Contre le Cancer, ed. *Pédiatrie II. Neuroblastome et médulloblastome*. Paris: John Libbey EUROTEXT, vol.10: 167–222.

Doz F, Alapetite C, Chastagner P, Choux M, Gaboriaud G, Pontvert D, Sarradet A (1998) Standards, options et recommandations. Médulloblastome de l'enfant. In: Fédération Nationale des Centres de Lutte Contre le Cancer, ed. *Recommandations pour la pratique clinique en cancérologie* [CD-Rom]. Paris: FNCLCC, John Libbey EUROTEXT.

#### 3.2.2 Neuroblastoma

Bergeron C, Blanc-Vincent MP, Bouvier R, Combaret V, Gauthier F, Giammarile F, Hartmann O, Mathieu P, Michon J, Neuenschwander S, Philip T, Plantaz D, Ranchère D, Rubie H, Thiesse P, Fédération Nationale des Centres de Lutte Contre le Cancer, Société Française d'Oncologie Pédiatrique (1999) Standards, options et recommandations pour le neuroblastome de l'enfant. In: Fédération Nationale des Centres de Lutte Contre le Cancer, ed. *Pédiatrie II. Neuroblastome et médulloblastome*. Paris: John Libbey EUROTEXT, vol.10: 1–165.

Bergeron C, Blanc-Vincent MP, Bouvier R, Combaret V, Gauthier F, Giammarile F, Hartmann O, Mathieu P, Michon J, Neuenschwander S, Philip T, Plantaz D, Ranchère D, Rubie H, Theisse P (1998) Standards, options et recommandations pour la neuroblastome de l'enfant. In: Fédération Nationale des Centres de Lutte Contre le Cancer, ed. *Recommandations pour la pratique clinique en cancérologie* [CD-Rom]. Paris: FNCLCC, John Libbey EUROTEXT

### 4. GOOD PRACTICE AND TREATMENT

### 4.1 Treatment

#### 4.1.1 Cytotoxic drugs

Crauste-Manciet S, Faure P, Latour JF, Madelaine-Chambrin I, Saulnier JL, Canal P, Cazin JL, Robert J, Husson MC and Montagnier-Pétrissans C (1998) Centre National Hospitalier d'Information sur le Médicament (CNHIM). Médicaments anticancéreux. In: Fédération Nationale des Centres de Lutte Contre le Cancer, ed. *Recommandations pour la pratique clinique en cancérologie* [CD-Rom]. Paris: FNCLCC, John Libbey EUROTEXT

Crauste-Manciet S, Faure P, Latour JF, Madelaine-Chambrin I and Saulnier JL (1995) Standards, options et recommandations pour l'utilisation pratique des anticancéreux. *Bull Cancer* **82**: 319s–452s

#### 4.1.2 Antiemetic therapy

Rivière A, Beckendorf V, Blanc-Vincent MP, Bonneterre J, Bui NB, Catimel G, Cupissol D, Fargeot P, Hecquet B, Krakowski I, Madelaine-Chambrin I, Mihura J, Roche H, Ruffié P, Schell M, Schneider M and Philip T (1998) Standards, options et recommandations pour l'utilisation des antiémétiques en cancérologie. In: Fédération Nationale des Centres de Lutte Contre le Cancer, ed. *Recommandations pour la pratique clinique en cancérologie* [CD-Rom]. Paris: FNCLCC, John Libbey EUROTEXT

Rivière A, Beckendorf V, Bonneterre J, Bui NB, Catimel G, Cupissol D, Fargeot P, Hecquet B, Krakowski I, Madelaine-Chambrin I, Mihura J, Roche H, Ruffié P, Schneider M and Philip T (1995) Standards, options et recommandations pour l'utilisation des antiémétiques en cancérologie. *Bull Cancer* **82**: 453s–485s

#### 4.1.3 Haematological growth factors

Blay JY, Le Cesne A, Blanc-Vincent MP, Fervers B, Latour JF and Philip T (1998) Standards, options et recommandations pour l'utilisation des facteurs de croissance hématopoïétique en cancérologie. In: Fédération Nationale des

© 2001 FNCLCC

Centres de Lutte Contre le Cancer, ed. *Recommandations pour la pratique clinique en cancérologie* [CD-Rom]. Paris: FNCLCC, John Libbey EUROTEXT

Blay JY, Fervers B, Latour JF and Philip T (1995) Standards, options et recommandations pour l'utilisation des facteurs de croissance hématopoïétique en cancérologie. *Bull Cancer* **82**: 487s–508s

### 4.1.4 Erythropoietin

Spaëth D, Marchal C, Bataillard A and Blanc-Vincent MP (1999) Eléments de la mise à jour 1999 des Standard, Options, Recommandations pour l'utilisation de l'érythropoïétine en cancérologie. *Bull Cancer* **86**(7–8): 631–639

Spaëth D, Marchal C and Blanc-Vincent MP (1998) Standards, options et recommandations pour l'utilisation de l'érythropoïetine en cancérologie. *Bull Cancer* **85**: 337–346

Spaëth D, Marchal C and Blanc-Vincent MP (1998) Standards, options et recommandations pour l'utilisation de l'érythropoïetine en cancérologie. In: Fédération Nationale des Centres de Lutte Contre le Cancer, ed. *Recommandations pour la pratique clinique en cancérologie* [CD-Rom]. Paris: FNCLCC, John Libbey EUROTEXT

### 4.1.5 Pain management

Krakowski I, Boureau F, Falcoff H, Gestin Y, Goldberg J, Guillain H, Jaulmes F, Lakdja F, Larue F, Magnet M, Meynadier J, Poulain P, Pozzo di Borgo C, Rebattu P, Salamagne M, Serrie A, Schach R, Trechot P, Verdie JC, Fédération Nationale des Centres de Lutte Contre le Cancer, Société Francophone d'Etude de la Douleur (1998) Recommandations pour une bonne pratique dans la prise en charge de la douleur du cancer chez l'adulte et l'enfant. In: Fédération Nationale des Centres de Lutte Contre le Cancer, ed. *Recommandations pour la pratique clinique en cancérologie* [CD-Rom]. Paris: FNCLCC, John Libbey EUROTEXT

Krakowski I, Falcoff H, Gestin Y, Goldberg J, Guillain H, Jaulmes F, Lakdja F, Larue F, Magnet M, Meynadier J, Poulain P, Pozzo di Borgo C, Rebattu P, Salamagne M, Serrie A, Schach R, Trechot P and Verdie JC (1996) Recommandations pour une bonne pratique dans la prise en charge de la douleur du cancer chez l'adulte et l'enfant. *Bull Cancer* **83**: 9s–79s

Krakowski I, Falcoff H, Gestin Y, Goldberd Y, Guillain H, Jaulmes F, Lakdja F, Larue F, Magnet M, Meynadier J, Poulain P, Pozzo di Borgo C, Rebattu P, Salamagen M, Serrie A, Schach R, Trechot P and Verdie JC (1995) Recommandations pour une bonne pratique dans la prise en charge de la douleur chez l'adulte et l'enfant. *Bull Cancer* **82**(suppl 4): 245s–315s

### 4.1.6 Nutritional support in haemato-oncology

Desport JC, Blanc-Vincent MP, Gory-Delabaere G, Bachmann P, Béal J, Benamouzig R, Colomb V, Kere D, Melchior JC, Nitenberg G, Raynard B, Schneider S and Senesse P (2000) Standards, options et recommandations (SOR) pour l'utilisation des médicaments orexigènes en cancérologie. *Bull Cancer* **87**: 315–328

Nitenberg G, Blanc-Vincent MP and Philip T (2000) Standards, options et recommandations (SOR): assistance nutritionnelle en onco-hématologie [éditorial]. *Bull Cancer* **87**: 311–313

### 4.1.7 Complimentary medicine

Schraub S (1998) Standards, options et recommandations concemant les médecines parallèles. In: Fédération Nationale des Centres de Lutte Contre le Cancer, ed. *Recommandations pour la pratique clinique en cancérologie* [CD-Rom]. Paris: FNCLCC, John Libbey EUROTEXT

### 4.2 Infection and cancer

### 4.2.1 General principles

Viot M, Béal J, Biron P, Blanc-Vincent MP, Boutard P, Bussy V, Crokaert F, Escande MC, Fuhrman C, Lesimple T, Pény J, Pottecher B, Raveneau J, Senet JM and Thyss A (2000) Infection et cancer: notions générales et questions actuelles. *Presse Med* (in Press)

Viot M, Béal J Biron P, Blanc-Vincent MP, Boutard P, Bussy V, Crokaert F, Escande MC, Fuhrman C, Lesimple I, Pény J, Pottecher B, Raveneau J, Senet JM and Thyss A (1999) Standards, options et recommandations. Infection et cancer: notions générales et questions actuelles. In: Fédération Nationale des Centres de Lutte Contre le Cancer, ed. *Infection et cancer*, vol.8.Paris: John Libbey EUROTEXT: Paris, pp 9–36

Viot M, Béal J, Biron P, Blanc-Vincent MP, Boutard P, Bussy V, Crokaert F, Escande MC, Fuhrman C, Lesimple T, Pény J, Pottecher B, Raveneau J, Senet JM and Thyss A (1998) Standards, options et recommandations. Infection et cancer: notions générales et questions actuelles. In: Fédération Nationale des Centres de Lutte Contre le Cancer, ed. *Recommandations pour la pratique clinique en cancérologie* [CD-Rom]. Paris: FNCLCC, John Libbey EUROTEXT

### 4.2.2 Management of short duration neutropenia

Biron P, Fuhrman C, Escande MC, Blanc-Vincent MP, Crokaert F, Béal J, Bussy V, Lesimple T, Pottecher B, Raveneau J, Senet JM and Viot M (1999) Standards, options et recommandations pour la prise en charge des neutropénies courtes. In: Fédération Nationale des Centres de Lutte Contre le Cancer, ed. *Infection et cancer*. Paris: John Libbey EUROTEXT, vol. 8:129–179

Biron P, Fuhrmann C, Escande MC, Blanc-Vincent MP, Crokaert F, Béal J, Bussy V, Lesimple T, Pottecher B, Raveneau J, Senet JM and Viot M (1998) Standards, options et recommandations pour la prise en charge des neutropénies courtes. *Bull Cancer* **85**: 695–711

Biron P, Fuhrman C, Escande MC, Blanc-Vincent MP, Crokaert F, Béal J, Bussy V, Lesimple T, Pottecher B, Raveneau J, Senet JM and Viot M (1998) Infection et cancer: standards, options et recommandations pour la prise en charge des neutropénies courtes. In: Fédération Nationale des Centres de Lutte Contre le Cancer, ed. *Recommandations pour la pratique clinique en cancérologie* [CD-Rom]. Paris: FNCLCC, John Libbey EUROTEXT.

### 4.2.3 Good practice in blood culture

Bussy V, Escande MC, Fuhrman C, Boineau F, Blanc-Vincent MP, Béal J, Biron P, Crokaert F, Lesimple T, Pottecher B, Raveneau J, Senet JM and Viot M (2000) Standards, Options et Recommandations pour une bonne pratique des hémocultures en cancérologie. *Presse Med* (In press)

Bussy V, Escande MC, Fuhrman C, Boineau F, Blanc-Vincent MP, Béal J, Biron P, Crokaert F, Lesimple T, Pottecher B, Raveneau J, Senet JM and Viot M (1999) Standards, options et recommandations pour une bonne pratique des hémocultures en cancérologie. In: Fédération Nationale des Centres de Lutte Contre le Cancer, ed. *Infection et cancer*. Paris: John Libbey EUROTEXT, vol.8: 37–62

Bussy V, Escande MC, Fuhrman C, Boineau F, Blanc-Vincent MP, Béal J, Biron P, Crokaert F, Lesimple T, Pottecher B, Raveneau J, Senet JM and Viot M (1998) Infection et cancer: standards, options et recommandations pour une bonne pratique des hémocultures en cancérologie. In: Fédération Nationale des Centres de Lutte Contre le Cancer, ed. *Recommandations pour la pratique clinique en cancérologie* [CD-Rom]. Paris: FNCLCC, John Libbey EUROTEXT

### 4.2.4 Infectious complications associated with the administration of interleukin-2

Escande MC, Blanc-Vincent MP, Lesimple T, Bussy V, Béal J, Crokaert F, Biron P, Viot M, Fuhrman C, Pottecher B, Senet JM and Raveneau J (1999) Standards, options et recommandations: prévention, diagnostic et traitement des complications infectieuses liées à l'administration intraveinouco d'interleukine 2. In: Fédération Nationale des Centres de Lutte Contre le Cancer, ed. *Infection et cancer*. Paris: John Libbey EUROTEXT, vol.8: 237–260

Escande MC, Blanc-Vincent MP, Lesimple T, Bussy V, Béal J, Crokaert F, Biron P, Viot M, Fuhrman C, Pottecher B, Senet JM and Raveneau J (1998) Infection et cancer: standards, options et recommandations pour la prévention, le diagnostic et le traitement des complications infectieuses liées à l'administration intraveineuse d'interleukine 2. In: Fédération Nationale des Centres de Lutte Contre le Cancer, ed. *Recommandations pour la pratique clinique en cancérologie* [CD-Rom]. Paris: FNCLCC, John Libbey EUROTEXT

### 4.2.5 Infections associated with in-dwelling lines

Lesimple T, Béal J, Escande MC, Lion C, Blanc-Vincent MP, Bussy V, Biron P, Pottecher B, Crokaert F, Senet JM, Fuhrman C, Raveneau J and Viot M (1999) Standards, options et recommandations pour la prévention, le diagnostic et le traitement des infections liées aux voies veineuses en cancérologie. In: Fédération Nationale des Centres de Lutte Contre le Cancer, ed. *Infection et cancer*. Paris: John Libbey EUROTEXT, vol.8: 63–128

Lesimple T, Béal J, Escande MC, Lion C, Blanc-Vincent MP, Bussy V, Biron P, Pottecher B, Crokaert F, Senet JM, Fuhrman C, Raveneau J and Viot M (1998) Infection et cancer: standards, options et recommandations pour la prévention, le diagnostic et le traitement des infections liées aux voies veineuses en cancérologie. In: Fédération Nationale des Centres de Lutte Contre le Cancer, ed. *Recommandations pour la pratique clinique en cancérologie* [CD-Rom]. Paris: FNCLCC, John Libbey EUROTEXT

© 2001 FNCLCC

### 4.2.6 Candidiasis

Senet JM, Herbrecht R, Blanc-Vincent MP, Béal J, Biron P, Bussy V, Crokaert F, Lesimple T, Escande MC, Fuhrman C, Pottecher B, Raveneau J and Viot M (1999) Standards, options et recommandations pour la prévention, le diagnostic et le traitement des candidoses en cancérologie. In: Fédération Nationale des Centres de Lutte Contre le Cancer, ed. *Infection et cancer*. Paris: John Libbey EUROTEXT, vol. 8: 181–236

Senet JM, Herbrecht R, Blanc-Vincent MP, Béal J, Biron P, Bussy V, Crokaert F, Lesimple T, Escande MC, Fuhrman C, Pottecher B, Raveneau J and Viot M (1998) Infection et cancer: standards, options et recommandations pour la prévention, le diagnostic et le traitement des candidoses en cancérologie. In: Fédération Nationale des Centres de Lutte Contre le Cancer, ed. *Recommandations pour la pratique clinique en cancérologie* [CD-Rom]. Paris: FNCLCC, John Libbey EUROTEXT

### 4.2.7 Nosocomial infections

Pottecher B, Herbrecht R, Blanc-Vincent MP, Bussy Malgrange V, Escande MC, Fuhrmann C, Crokaert F, Gory-Delabaere G, Senet JM, Lesimple T, Raveneau J, Béal J, Biron P and Viot M (2000) Standards, Options et Recommandations (SOR) pour la surveillance et la prévention des infections nosocomiales en cancérologie. *Bull Cancer* **87**: 557–591

### 4.3 Good diagnostic and clinical practice

### 4.3.1 Good practice in pathology

Arnould L, Fiche M, Blanc-Vincent MP, Le Doussal V, Zafrani B, Gory-Delabaere G, Brifford M, Vielh P and Voigt JJ (2000) Standards, options et recommandations pour la rédaction d'un compterendu d'anatomie et cytologie pathologiques en cancérologie. *Bull Cancer* **87**: 159–171

Voigt JJ and Philip T (2000) Standards, options et recommandations (SOR): anatomie et cytologie pathologiques en cancérologie [éditorial]. *Bull Cancer* **87** 155–157

### 4.3.2 Good practice in tumour markers

Basuyau JP, Blanc-Vincent MP, Bidart JM, Daver A, Deneux L, Eche N, Gory-Delabaere G, Pichon MF and Riedinger JM (2000) Standards, Options et Recommandations: marqueurs tumoraux sériques et cancer du sein. *Bull Cancer* **87**(10): A paraître

### 4.3.3 Good practice in chemotherapy

Héron JF, Fervers B, Latour JF, Sommelet D, Chauvergne J and Philip T (1998) Standards, options et recommandations pour une bonne pratique en chimiothérapie. In: Fédération Nationale des Centres de Lutte Contre le Cancer, ed. *Recommandations pour la pratique clinique en cancérologie* [CD-Rom]. Paris: FNCLCC, John Libbey EUROTEXT

Héron JF, Fervers B, Latour JF, Sommelet D, Chauvergne J and Philip T (1995) Standards, options et recommandations pour une bonne pratique en chimiothérapie. *Bull Cancer* **82**: 823–834

### 4.3.4 Good practice in radiotherapy

Bey P, Gaboriaud G, Peiffert D, Aletti P and Cosset JM (1995) Standards, options et recommandations pour une bonne pratique en radiothérapie externe et en curiethérapie en cancérologie. *Bull Cancer* **82**: 811–822

Bey P, Gaboriaud G, Peiffert D, Aletti P and Cosset JM (1998) Standards, options et recommandations pour une bonne pratique en radiothérapie externe et en curiethérapie en cancérologie. In: Fédération Nationale des Centres de Lutte Contre le Cancer, ed. *Recommandations pour la pratique clinique en cancérologie* [CD-Rom]. Paris: FNCLCC, John Libbey EUROTEXT

### 4.3.5 Good practice in surgery

Dauplat J, Depadt G, Abbes M, Bobin JY, Carolus JM, Chardot C, Durand JC, Fondrinier E, Fraisse J, Guillemin F, Janser JC, Julien JP, Lallement M, Lasser P, Laurent JC, Lorimier G, Michel G, Pujol H, Rauch P, Rouanet P, Saint-Aubert B, Stockle E and Verhaeghe JL (1995) Standards, options et recommandations pour une bonne pratique en chirurgie cancérologique. *Bull Cancer* **82**: 795–810

Dauplat J, Depadt G, Abbes M, Bobin JY, Carolus JM, Chardot C, Durand JC, Fondrinier E, Fraisse J, Guillemin F, Janser JC, Julien JP, Lallement M, Lasser P, Laurent JC, Lorimier G, Michel G, Pujol H, Rauch P, Rouanet P, Saint-Aubert B, Stockle E and Verhaeghe JL (1998) Standards, options et recommandations pour une bonne pratique en chirurgie cancérologique. In: Fédération Nationale des Centres de Lutte Contre le Cancer, ed. *Recommandations pour la pratique clinique en cancérologie* [CD-Rom]. Paris: FNCLCC, John Libbey EUROTEXT

### 4.3.6 Good practice in multidisciplinary care

Bey P, Abbatucci JS, Chardot C, Farsi F, Fervers B and Philip T (1998) Standards, options et recommandations pour une bonne pratique dans l'organisation pluridisciplinaire de la cancérologie. In: Fédération Nationale des Centres de Lutte Contre le Cancer, ed. *Recommandations pour la pratique clinique en cancérologie* [CD-Rom]. Paris: FNCLCC, John Libbey EUROTEXT

Chardot C, Fervers B, Bey P, Abbatucci JS and Philip T (1995) Standards, options et recommandations pour une bonne pratique dans l'organisation pluridisciplinaire de la cancérologie. *Bull Cancer* **82**: 780–794

### 4.3.7 Good practice in nuclear medicine

Maublant J, Fervers B, Herry JY, Lumbroso JD, Parmentier C, Pasquier J and Sulman C (1998) Standards, options et recommandations pour une bonne pratique de la médecine nucléaire in vivo en cancérologie. *Revue de l'ACOMEN* **4**: 63–76

Maublant J, Fervers B, Herry JY, Lumbroso JD, Parmentier C, Pasquier J and Sulman C (1998) Standards, options et recommandations pour une bonne pratique de la médecine nucléaire in vivo en cancérologie. In: Fédération Nationale des Centres de Lutte Contre le Cancer, ed. *Recommandations pour la pratique clinique en cancérologie* [CD-Rom]. Paris: FNCLCC, John Libbey EUROTEXT

### 4.3.8 Good practice in psycho-oncology

Saltel P, de Raucourt D, Derzelle M, Desclaux B, Fresco R, Gauvain Piquard A, Genot JY, Landry N, Lanzarotti C, Lehmann A, Machavoine JL, Marx G, Oppenheim D and Salimpour A (1998) Standards, options et recommandations pour une bonne pratique en psycho-oncologie. In: Fédération Nationale des Centres de Lutte Contre le Cancer, ed. *Recommandations pour la pratique clinique en cancérologie* [cédérom]. Paris: FNCLCC, John Libbey EUROTEXT

Saltel P, de Raucourt D, Derzelle M, Desclaux B, Fresco R, Gauvain Piquard A, Genot JY, Landry N, Lanzarotti C, Lehmann A, Machavoine JL, Marx G, Oppenheim D and Salimpour A (1995) Standards, options et recommandations pour une bonne pratique en psycho-oncologie. *Bull Cancer* **82**: 847–864

### 4.3.9 Good practice in imaging

Stines J, Bey P, Bertrand AF, Cambier L, Carsin A, Collay R, Delignette A, Dilhuydy MH, Hagay C, Humeau F, Kaemmerlen P, Masselot J, Netter E, Plantet MM, Sigal R, Tardivon A, Thiesse P, Tournemaine N, Troufleau P and Vanel D (1998) Standards, options et recommandations pour une bonne pratique de l'imagerie radiologique en cancérologie. In: Fédération Nationale des Centres de Lutte Contre le Cancer, ed. *Recommandations pour la pratique clinique en cancérologie* [CD-Rom]. Paris: FNCLCC, John Libbey EUROTEXT

Stines J, Bertrand AF, Cambier L, Carsin A, Collay R, Delignette A, Dilhuydy MH, Hagay C, Humeau F, Kaemmerten P, Masselot J, Netter E, Plantet MM, Sigal R, Tardivon A, Thiesse P, Tournemaine N, Troufleau P and Vanel D (1995) Standards, options et recommandations pour une bonne pratique de l'imagerie radiologique en cancérologie. *Bull Cancer* **82**: 835–845

### 4.3.10 Good practice in dental care

Maire F, Borowski B, Collangettes D, Farsi F, Guichard M, Gourmet R and Kreher P (1999) Standards, Options et Recommandations pour une bonne pratique odontologique en cancérologie. *Bull Cancer* **86**(7–8): 640–665

### 4.3.11 Good practice in dietetics

#### 4.3.11.1 Good practice in dietetics for cancers of the upper aerodigestive tract

Meuric J, Garabige V, Blanc-Vincent MP, Lallemand Y and Bachmann P (1999) Bonnes pratiques pour la prise en charge diététique des patients atteints de cancer des voies aérodigestives supérieures. *Bull Cancer* **86**(10): 843–854

#### 4.3.11.2 Good practice in dietetics: nutrition and its evaluation

Duguet A, Bachmann P, Lallemand Y and Blanc-Vincent MP (1999) Bonnes pratiques diététiques en cancérologie: dénutrition et évaluation nutritionnelle. *Bull Cancer* **86**(12): 997–1016

*© 2001 FNCLCC*

### *4.3.12 Genetic counselling*

Eisinger F, Thouvenin D, Bignon YJ, Cuisenier J, Feingold J, Hoerni B, Lasset C, Lyonnet D, Maraninchi D, Marty M, Mattei JF, Sobol H, Maugard-Louboutin C, Nogues C, Pujol H and Philip T (1995) Réflexions sur l'organisation des consultations d'oncogénétique (première étape vers la publication de bonnes pratiques cliniques). *Bull Cancer* **82**: 865–878

Eisinger F, Thouvenin D, Bignon YJ, Cuisenier J, Feingold J, Hoerni B, Lasset C, Lyonnet D, Maraninchi D, Marty M, Mattei JF, Sobol H, Maugard-Louboutin C, Nogues C, Pujol H and Philip T (1998) Standards, options et recommandations. Réflexions sur l'organisation des consultations d'oncogénétique (première étape vers la publication de bonnes pratiques cliniques). In: Fédération Nationale des Centres de Lutte Contre le Cancer, ed. *Recommandations pour la pratique clinique en cancérologie* [CD-Rom]. Paris: FNCLCC, John Libbey EUROTEXT

### *4.3.13 Nurse delivered chemotherapy*

Teller AM, Bonnet C, Chalençon J, Deprados M, Doutre-Roussel D, Ehrhart M, Farsi F, Guicherd MF, Mathos G, Maurice F, Schlupp C, Schverzenzer N, Talon A and Titeux A (1998) Standards, options et recommandations: Bonne pratique des soins infirmiers en chimiothérapie anticancéreuse. In: Fédération Nationale des Centres de Lutte Contre le Cancer, ed. *Recommandations pour la pratique clinique en cancérologie* [CD-Rom]. Paris: FNCLCC, John Libbey EUROTEXT

Teller AM, Bonnet C, Chalençon J, Deprados M, Doutre-Roussel D, Ehrhart M, Guicherd MF, Mathos G, Maurice F, Schlupp C, Schverzenzer N, Talon A and Titeux A (1995) *Standards, options et recommandations: Bonne pratique des soins infirmiers en chimiothérapie anticancéreuse*. Paris: Expansion Scientifique Française, FNCLCC

### 4.4 Physical and social rehabilitation

### *4.4.1 Guide « prévoir demain »*

Daly-Schveitzer N, Dilhuydy JM, Azadian-Boulanger G, Blanc-Vincent MP, Brugère J, Chollet A, Couette J, Decuypère ML, Desclaux B, Dessena J, Devinat M, Dumortier A, Eyffred M, Faucher A, Ladaique P, Laplante P, Le Guen C, Machet C, May Levin F, Minier JF, Monira MH, Monticelli J, Netter E, Pommier P, Romieu G and Switsers O (1998) Standards, options et recommandations. Prévoir demain: guide pour la réinsersion des patients traités pour cancers. In: Fédération Nationale des Centres de Lutte Contre le Cancer, ed. *Recommandations pour la pratique clinique en cancérologie* [CD-Rom]. Paris: FNCLCC, John Libbey EUROTEXT

### 4.5 Epidemiology

### *4.5.1 Epidemiology of childhood cancers*

Benhamou E, Laplanche A and Philip T (1998) Epidémiologie descriptive des cancers de l'enfant (zéro à 14 ans) en France. In: Fédération Nationale des Centres de Lutte Contre le Cancer, ed. Recommandations pour la pratique clinique en cancérologie [cédérom]. Paris: FNCLCC, John Libbey EUROTEXT

### *4.5.2 Epidemiology of Cancer in the adult*

Estève J, Benhamou E, Hill C, Laplanche A and Philip T (1998) Epidémiologie descriptive des cancers de l'adulte en France. In: Fédération Nationale des Centres de Lutte Contre le Cancer, ed. Recommandations pour la pratique clinique en cancérologie [CD-Rom]. Paris: FNCLCC, John Libbey EUROTEXT

© 2001 FNCLCC

